# Presynaptic Mechanisms of l-DOPA-Induced Dyskinesia: The Findings, the Debate, and the Therapeutic Implications

**DOI:** 10.3389/fneur.2014.00242

**Published:** 2014-12-15

**Authors:** M. Angela Cenci

**Affiliations:** ^1^Basal Ganglia Pathophysiology Unit, Department of Experimental Medical Science, Lund University, Lund, Sweden

**Keywords:** neuroplasticity, neuropharmacology, neuropsychiatry, neurovascular unit, movement disorders, dystonia, basal ganglia, monoamines

## Abstract

The dopamine (DA) precursor l-DOPA has been the most effective treatment for Parkinson’s disease (PD) for over 40 years. However, the response to this treatment changes with disease progression, and most patients develop dyskinesias (abnormal involuntary movements) and motor fluctuations within a few years of l-DOPA therapy. There is wide consensus that these motor complications depend on both pre- and post-synaptic disturbances of nigrostriatal DA transmission. Several presynaptic mechanisms converge to generate large DA swings in the brain concomitant with the peaks-and-troughs of plasma l-DOPA levels, while post-synaptic changes engender abnormal functional responses in dopaminoceptive neurons. While this general picture is well-accepted, the relative contribution of different factors remains a matter of debate. A particularly animated debate has been growing around putative players on the presynaptic side of the cascade. To what extent do presynaptic disturbances in DA transmission depend on deficiency/dysfunction of the DA transporter, aberrant release of DA from serotonin neurons, or gliovascular mechanisms? And does noradrenaline (which is synthetized from DA) play a role? This review article will summarize key findings, controversies, and pending questions regarding the presynaptic mechanisms of l-DOPA-induced dyskinesia. Intriguingly, the debate around these mechanisms has spurred research into previously unexplored facets of brain plasticity that have far-reaching implications to the treatment of neuropsychiatric disease.

Parkinson’s Disease (PD) is defined by a set of motor signs and symptoms that are caused by dopamine (DA) deficiency and respond well to dopaminergic therapies. Accordingly, functional imaging studies have established a close link between the onset and severity of PD motor features and the loss of dopaminergic markers in the putamen (1, 2). Oral administration of the DA precursor, l-DOPA has provided the backbone of PD treatment for over 40 years [recently reviewed in Ref. (3, 4)]. However, this treatment leads to complications.

After a few years of l-DOPA pharmacotherapy, most PD patients will exhibit a shorter motor response to each medication dose (“wearing-off fluctuation”), often associated with choreiform abnormal involuntary movements (AIMs) that appear when plasma and brain levels of l-DOPA are high (“peak-dose dyskinesias”) (Figure 1). More complex response patterns may also occur, for example, dyskinesias appearing when plasma l-DOPA levels rise or decline after each dose (“diphasic dyskinesia”), or abrupt fluctuations between a good antiparkinsonian response and a severe parkinsonian motor state (“unpredictable on–off fluctuations”) [reviewed in Ref. (3, 5)]. It has recently been established that oral l-DOPA therapy produces non-motor complications too, particularly, fluctuations in mood and cognitive performance (3, 6).

**Figure 1:**
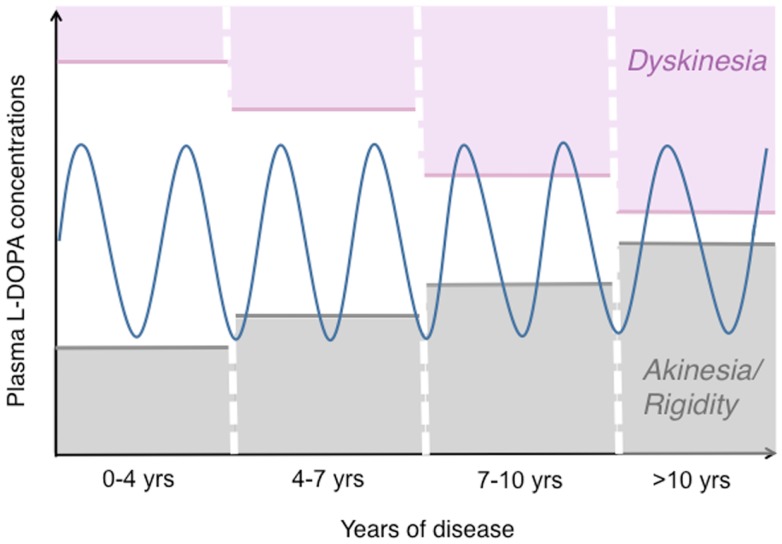
**The pattern of motor response to l-DOPA changes during the progression of PD**. This drawing illustrates how the therapeutic window of l-DOPA narrows during the progression of PD [based on (172, 173)]. While oral l-DOPA therapy achieves a stable symptomatic control during the first years, it causes motor fluctuations and dyskinesias in more advanced disease stages. Dyskinesias are most commonly associated with high plasma levels of l-DOPA (peak-dose LID), as shown here. The blue sinuous line represents peaks-and-troughs in plasma l-DOPA levels concomitant with oral l-DOPA therapy. The empty area at the centre represents the range of l-DOPA concentrations that induce relief of PD motor features without causing dyskinesia.

Factors associated with a higher incidence and/or early development of l-DOPA-induced dyskinesia (LID) include, l-DOPA dosage, severity and duration of PD (7, 8, 9), and a young age at PD onset [reviewed in Ref. (8, 9)]. Some autosomal recessive forms of PD also entail a high risk of LID (10), possibly because they share many features with young-onset idiopathic PD, in particular, a severe loss of DA neurons with relative preservation of non-dopaminergic systems and slow progression of Lewy-related brain pathology (11, 12). The reasons underlying a high risk for LID in young-onset PD patients have not been resolved, and several valid hypotheses have been put forward, including a faster DA turnover (13) or a larger potential for neuroplasticity in a younger brain (14). Moreover, the relative integrity of non-dopaminergic systems in younger subjects may contribute to a higher risk for LID. These systems may include corticostriatal and/or serotonergic projections, as will be discussed in this article.

## “Pre- or Post-Synaptic Mechanisms?” A Brief Historical Perspective

In the most typical cases, dyskinesias and motor fluctuations are temporally related to rises and declines in plasma l-DOPA levels (Figure 1). In advanced stages of PD, the same dosage of l-DOPA that is required to relieve parkinsonian features may also induce AIMs [reviewed in Ref. (3, 15) and schematically illustrated in Figure 1].

Whether this altered response pattern depends on presynaptic or post-synaptic changes in nigrostriatal DA transmission has been a matter of major debate. The presynaptic hypothesis, which prevailed in the 80s, held that the progressive degeneration of nigral neurons causes a loss of DA storage capacity in nigrostriatal nerve terminals (16). Under these conditions, l-DOPA would be immediately converted to DA by a variety of cells in the brain, and rapidly eliminated. Peak-dose LID and wearing-off fluctuations would thus be the clinical counterparts of swift rises and declines in central DA levels, respectively [reviewed in Ref. (17)].

During the 90s, the presynaptic hypothesis appeared to decrease in popularity as many investigators turned one’s attention to the post-synaptic consequences of DA depletion. The attention shift was prompted by studies in 6-hydroxydopamine (6-OHDA)-lesioned rats, which revealed striking effects of chronic l-DOPA treatment on the expression of GABA-biosynthetic enzymes, neuropeptides, and opioid precursors in striatal neurons (18). In addition, studies in PD patients revealed that the therapeutic window of apomorphine, a direct DA agonist, narrowed with the progression from a DOPA-naive to a DOPA-treated dyskinetic state (19). Because apomorphine acts independently of presynaptic nigrostriatal terminals, these results were used to suggest that altered signal-transduction mechanisms in striatal neurons are the main culprit of motor complications to PD therapy (19).

Presynaptic factors were brought back into the limelight by human positron emission tomography (PET) studies using the reversible D2 receptor ligand, [^11^C] raclopride to estimate DA release. This approach takes advantage of a competition between endogenous DA and [^11^C] raclopride for binding to D2 receptors. Increased DA levels in the striatum are thus seen as a reduction in [^11^C] raclopride binding potential compared to baseline values. Using this technique, De la Fuente Fernandez and colleagues showed that standard oral doses of l-DOPA caused larger swings in striatal DA levels in PD patients experiencing motor complications compared to patients with a stable response to treatment (20, 21). Moreover, Piccini and collaborators found a positive linear relationship between putaminal changes in [^11^C] raclopride binding and AIM scores “on” l-DOPA (22). These human studies provided a strong support to the presynaptic hypothesis of LID, and prompted a new wave of clinical and preclinical research aimed at shedding light on the mechanisms involved.

**Figure 2:**
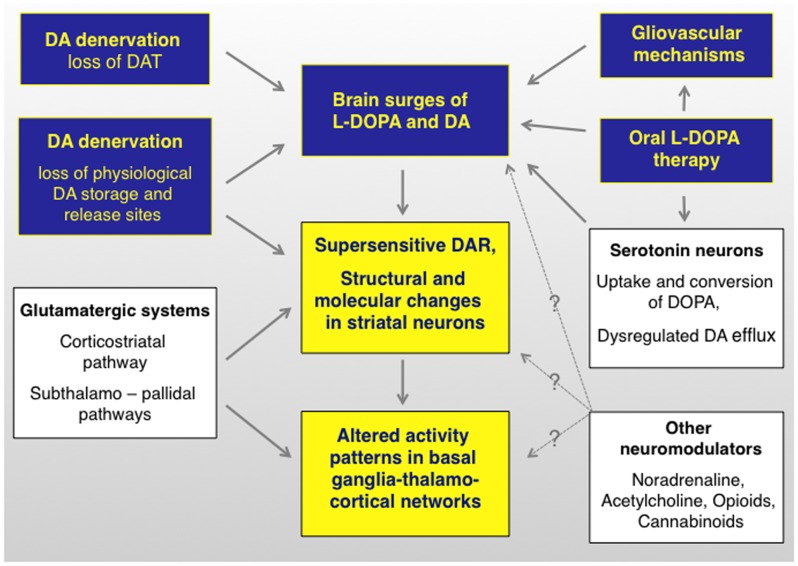
**l-DOPA-induced dyskinesia depends on both pre- and postsynaptic disturbances of DA transmission that are modulated by non-dopaminergic transmitter systems**. The term “presynaptic” refers to all factors that contribute to generating fluctuating levels of l-DOPA and DA in the brain (blue boxes). The term post-synaptic refers to mechanisms that occur at the level of dopaminoceptive cells (yellow boxes). Non-dopaminergic modulatory systems are shown in white boxes. It is not well understood how these systems modulate different levels of the pathophysiological cascade (hence the question marks). DAR, dopamine receptors. Studies supporting this pathophysiological cascade have been reviewed in Ref. (3, 27, 174, 175). An updated review on the presynaptic factors is presented in this article.

During the past 10 years, different groups of investigators have continued to debate on whether or not presynaptic factors can by themselves drive the development of LID (23, 24), and experimental evidence has been put forward to either support or reject this standpoint [cf., e.g., Ref. (25, 26)]. Because a disruption of presynaptic DA homeostasis will certainly have post-synaptic consequences (27) (Figure 2), this debate may appear artificially contentious at first glance. However, it is becoming clear that the relative weight of presynaptic versus post-synaptic mechanisms in generating the involuntary movements will condition the response to antidyskinetic interventions (28).

**Figure 3:**
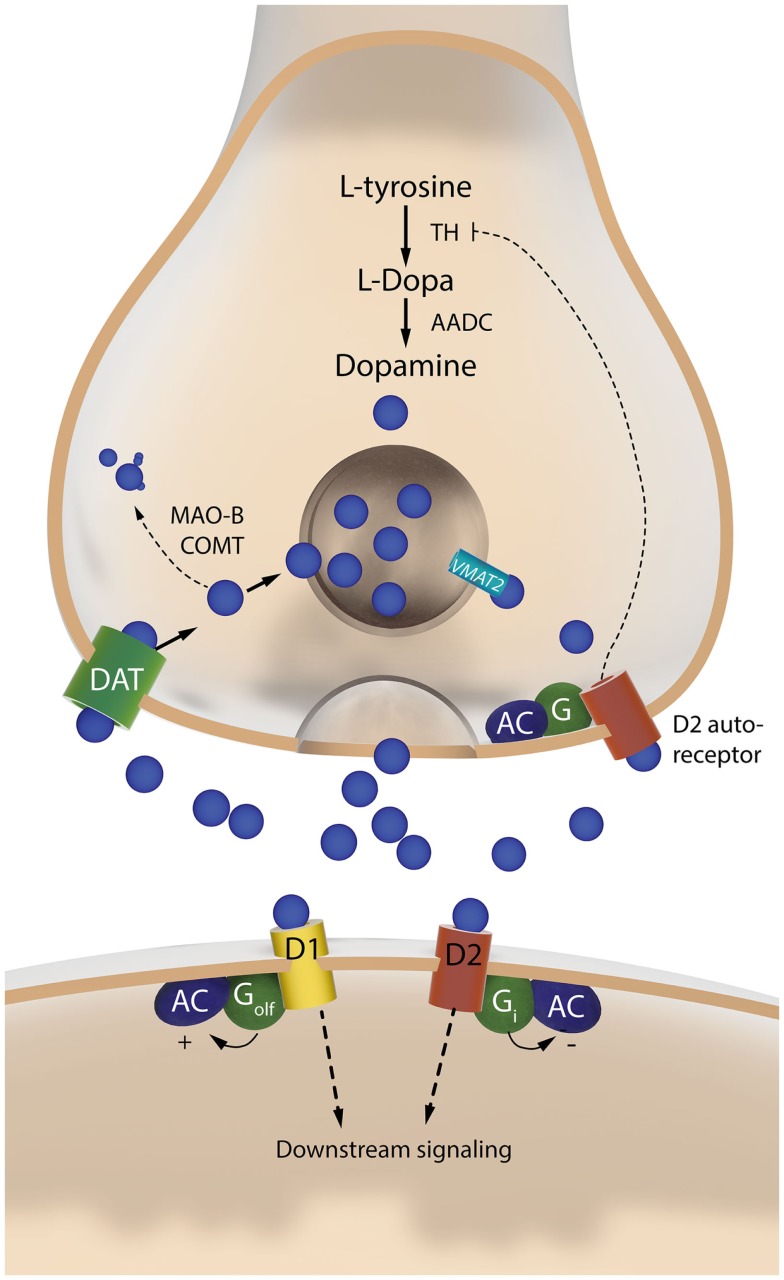
**The two sides of a dopaminergic synapse**. The drawing illustrates components of the nigrostriatal dopaminergic synapse that are discussed in this article. The presynaptic nigrostriatal terminal releases DA (blue circles), and regulates extracellular DA levels through several mechanisms: DA reuptake from the extracellular fluid (via the DAT), DA transport into synaptic vesicles (via VMAT-2), DA synthesis (which is subjected to autoregulatory control via presynaptic D2 receptors), and DA metabolism (via MAO-B and COMT). The post-synaptic neuron responds to DA via two main types of receptors. The D1 receptor is coupled to G_olf_ and activates c-AMP-dependent intracellular signaling pathways. The D2 receptor is coupled to G_i_ and inhibits the same pathways. AADC, aromatic L-amino acid decarboxylase; AC, adenylate cyclase; COMT, catechol-O-methyl-transferase; DAT, dopamine transporter; MAO-B, monoamine oxidase B; TH, tyrosine hydroxylase; VMAT-2, vesicular monoamine transporter 2.

This review article will summarize both the terms of the debate and the valuable research that has stemmed from it. Thanks to this research, conspicuous progress has been made toward understanding specific players on the “presynaptic side” of the cascade (summarized in Figures 2 and 3).

## Nigrostriatal DA Denervation and l-DOPA Dosage are Critical to LID

Clinical observations suggest that the loss of nigrostriatal DA neurons plays an important role in the development of LID (8, 9, 29). But PD has a complex pathology, and it is difficult to demonstrate the causal link between dopaminergic denervation and LID in human studies. This type of information can however be inferred from experimental models of the movement disorder.

In all the most common animal models of PD–LID, the loss of nigrostriatal neurons is obtained using specific neurotoxins. 6-Hydroxydopamine (6-OHDA) and 1-methyl-4-phenyl-1,2,3,6-tetrahydropyridine (MPTP) have been the most commonly used toxins in rodents and non-human primate species, respectively. In all the current animal models, the AIMs induced by l-DOPA mimic the peak-dose variant of human LID [reviewed in Ref. (30)].

Non-human primate studies examining the relationship between LID and extent of nigrostriatal DA lesion have been sparse and, at first glance, conflicting. A seminal study in MPTP-lesioned macaques reported that therapeutic doses of l-DOPA produced dyskinesia only in monkeys having ≥95% striatal DA loss (31). Accordingly, a study in MPTP-lesioned marmosets reported that only animals with >85% striatal DA loss developed choreoathetoid dyskinesias with therapeutic doses of l-DOPA, and that the most severely parkinsonian animals displayed the most severe LID (32). However, studies in squirrel monkeys reported choreoathetoid dyskinesias in animals with partial striatal DA denervation (33), and even in intact animals treated with a therapeutic l-DOPA regimen (15 mg/kg twice daily for 2 weeks) (34). Furthermore, intact macaque monkeys were reported to develop choreoathetoid dyskinesias if treated with very high doses of l-DOPA (80 mg/kg/day for 13 weeks) (35). Thus, the impact of nigrostriatal DA denervation on the susceptibility to LID differs between non-human primate species, some of which can develop involuntary movements even in the absence of dopaminergic denervation, if given sufficiently high doses of l-DOPA.

**Figure 4:**
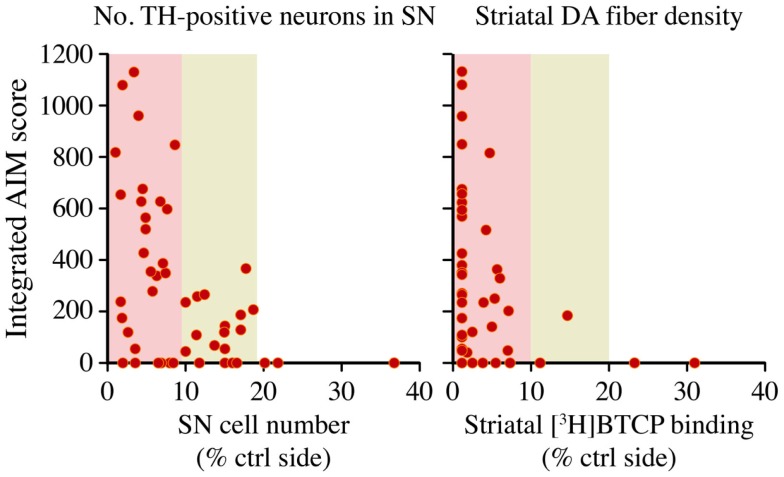
**A large nigrostriatal DA lesion is necessary but not sufficient for therapeutic l-DOPA doses to induce dyskinesia**. Rats sustained unilateral nigrostriatal DA lesions of varying severity, and were then treated with l-DOPA (6 mg/kg/day) for 4 weeks. Diagrams plot the animals cumulative Abnormal Involuntary Movement (AIM) scores (*y* axis) on presynaptic markers of DA neuron integrity, that is, tyrosine hydroxylase- positive cells in the substantia nigra (SN) or striatal innervation density, estimated with DAT radioligand binding using [^3^H]-BTCP. Data collected on the side ipsilateral to the lesion are expressed as a percentage of the values on the contralateral (ctrl) intact side. With either measure, AIM scores were found to occur only in animals that had lost more than 80% of presynaptic dopaminergic markers, and maximally severe AIMs occurred only when this loss exceeded 90%. Note however that some of the completely DA-denervated animals did not develop any dyskinesia. The dataset is derived from Ref. (36).

The largest rodent study addressing the relationship between nigrostriatal DA lesion and LID severity is the one by Winkler and colleagues (36). In this study, rats sustained partial or complete lesions of the nigrostriatal pathway, and were then treated with l-DOPA at a low therapeutic dose (6 mg/kg/day) for 4 weeks. Only rats with >80% loss of striatal dopamine transporter (DAT) or nigral DA neurons developed dyskinetic behaviors, and involuntary movements of maximal severity occurred only in the subgroup exhibiting >90% loss of dopaminergic markers (36). However, some of the completely DA-denervated animals remained free from dyskinetic behaviors throughout the l-DOPA treatment period (Figure 4). Thus, although a large nigrostriatal DA lesion was necessary for l-DOPA to induce involuntary movements, the severe dopaminergic denervation was not by itself sufficient (36). A similar conclusion was reached by Bezard and collaborators in a study using MPTP-lesioned macaques (37). It should be added, however, that high doses of l-DOPA will induce dyskinesia in all animals exhibiting >90% loss of dopaminergic markers throughout the caudate-putamen, although the actual doses will vary depending on species [c.f. ≥25 mg/kg/day in the rat (38, 39) versus ≥3 mg/kg/day in mice (40, 41)].

In summary, the bulk of experimental data indicate that, if l-DOPA is given at a therapeutic dosage, involuntary movements develop only when the loss of DA afferents to the motor striatum exceeds a threshold level of 80–85%. Despite these large lesions, some animals will however remain free from LID during the chronic treatment. Intriguingly, these experimental observations are in keeping with the clinical experience, whereby a proportion of PD patients never develop dyskinesias during their lifetime exposure to l-DOPA (9). Autoradiographic studies of DAT binding in the post-mortem striatum have not detected a difference between dyskinetic and non-dyskinetic PD cases (42, 43), indicating that a severe dopaminergic denervation is not sufficient for some patients to develop LID. Thus, although presynaptic DA depletion predicts the risk of LID (29), the susceptibility to this therapy complication must also depend on additional factors. These factors are likely to include some of the mechanisms discussed in the following sections.

## Presynaptic Consequences of Nigrostriatal DA Denervation

The degeneration of nigrostriatal DA neurons in PD implies a severe depletion of the presynaptic compartment that physiologically converts l-DOPA to DA, releases DA in a regulated fashion, and clears DA from the extracellular space via high-affinity reuptake (Figure 3). The nigrostriatal system has a high capacity to mount compensatory mechanisms after partial lesions through, e.g., increased DA turnover, sprouting of residual DA terminals, and downregulation of the DAT [reviewed in Ref. (15, 44)]. Accordingly, parkinsonian motor symptoms have been estimated to appear only after a loss of 50% nigral DA neurons and 70% striatal DA contents [reviewed in Ref. (15)]. Similar phenomena have been observed in 6-OHDA-lesioned rodents, where the compensatory capacity of the nigrostriatal system appears to break down only after a >70% loss of nigral DA neurons (45, 46).

The breakdown of presynaptic DA homeostasis predisposes to large fluctuations in central levels of DA upon treatment with l-DOPA. In a seminal microdialysis study, Abercrombie and collaborators showed that a peripheral injection of l-DOPA results in significantly higher extracellular DA levels in rats with large 6-OHDA lesions compared to intact animals (47). The l-DOPA-induced increase in striatal extracellular DA concentrations (ΔDA) was 30- to 80-fold larger in 6-OHDA-lesioned animals compared to intact controls (the striking difference being partly dependent on the lower baseline DA concentrations in lesioned animals) (47). This study also established a causal relationship between ΔDA and the lesion-induced loss of DAT. Indeed, combined treatment of intact rats with l-DOPA and nomifensine, a DAT inhibitor, produced increases in extracellular DA approaching the magnitude of those in 6-OHDA-lesioned animals (47). More recent studies have confirmed the crucial importance of DAT deficiency in determining large increases in extracellular DA “on” l-DOPA (48). However, these studies have also indicated that, when the nigrostriatal lesion is very severe, the magnitude of such increases depends on factors other than DAT deficiency. Thus, animals with less than 90% DA denervation exhibit a significant negative correlation between ΔDA and striatal DAT binding levels. However, in rats with >90% denervation, DAT levels no longer predict ΔDA (48). What factors may then condition the magnitude of ΔDA in animals with severe nigrostriatal DA lesions?

In addition to the loss of DAT, a severe degeneration of the nigrostriatal pathway inevitably entails a shift in the routes of l-DOPA metabolism from nigrostriatal DA neurons to other sites (15). The conversion of l-DOPA to DA is a one-step enzymatic reaction catalyzed by aromatic L-amino acid decarboxylase (AADC, also called DOPA decarboxylase, DDC) (Figure 3). This enzyme is expressed by catecholaminergic neurons (49), but also by astrocytes (50) and blood vessel-associated cells (51).

A seldom appreciated fact is that AADC and 5-hydroxytry ptophan decarboxylase (which synthetizes 5-hydroxytryptamine, serotonin) are the same enzyme (see, e.g., http://omim.org/entry/107930). Serotonin neurons therefore express relatively high levels of AADC, and they also express vesicular monoamine transporter 2 (VMAT-2), which packages DA into synaptic vesicles and protects it from rapid cytosolic degradation [reviewed in Ref. (15)] (cf. Figure 3). Although AADC and VMAT-2 also are expressed by noradrenergic neurons, these are unlikely to provide a major source of DA upon l-DOPA treatment, because the DA formed from l-DOPA in these neurons is rapidly converted to noradrenaline (NA) by the enzyme, dopamine-beta-hydroxylase.

Thus, serotonin neurons can both synthetize DA from l-DOPA, store the formed DA in synaptic vesicles, and release it in an activity-dependent manner. During the past few years, an abundant literature has documented that serotonin neurons indeed provide a source of DA release in l-DOPA-treated parkinsonian subjects. An intense debate has grown around the extent of this phenomenon and its significance to the occurrence of LID, as will be detailed in the following sections of this review. But before approaching this topic, we need to briefly consider the post-synaptic consequences of DA denervation, which are likely to be crucial to the development of LID.

## Post-Synaptic Consequences of Nigrostriatal DA Denervation

Although this article focuses on the presynaptic mechanisms of LID, it is important to keep in mind that a loss of nigrostriatal DA input also entails profound adaptations at the post-synaptic level (Figure 2). In particular, DA-denervating lesions cause pronounced molecular, physiological, and morphological changes in striatal neurons, as demonstrated by a large body of experimental literature, briefly reviewed below.

Already in the 70s, a deafferentation-induced supersensitivity of post-synaptic DA receptors was hypothesized to play a role in the development of LID (52). Today we know that this supersensitivity depends on complex changes in the signal-transduction properties of DA receptors. The changes include, an increased coupling efficiency of both D1 and D2 receptors to their corresponding G proteins, a large activation of downstream intracellular signaling molecules, changes in DA receptor trafficking, and also a striking activation of non-canonical signaling pathways [reviewed in Ref. (52–54)]. Gerfen and collaborators were the first to propose that the denervation-induced supersensitivity of D1 receptors leads to an activation of intracellular pathways that are not recruited under physiological conditions (55). In their seminal study (55), treatment of 6-OHDA-lesioned rats with D1 receptor agonists was found to cause a pronounced striatal activation of extracellular signal regulated kinases 1 and 2 (ERK1/2), a pathway traditionally associated with the stimulation of tyrosine-kinase or glutamate receptors, not G_s/olf_-coupled receptors (cf. Figure 2). A link between l-DOPA-induced ERK1/2 activation and the development of dyskinetic behaviors was later demonstrated in both rodent (40, 56–58) and non-human primate models of LID (40).

In addition to altered DA receptor-mediated signaling, an abnormal corticostriatal synaptic plasticity (59) and structural changes of striatal neurons associated with the progression of PD (60) predispose to a dyskinetic response to therapy. Post-mortem investigations of striatal tissue from PD patients have revealed conspicuous loss of spines and dendritic atrophy in medium-sized spiny neurons (61, 62). Similar phenomena have been found to occur in both rodent and non-human primate models of PD (63, 64). The results so far available indicate that treatment with l-DOPA does not normalize the dendritic structure of striatal neurons, but instead superimposes a new layer of changes that are associated with the development of dyskinetic behaviors (65–67).

It has been hypothesized that striatal dendritic atrophy has a major impact on the response to PD treatment favoring the emergence of complications because, “expecting a normal reaction to dopaminergic drugs under these circumstances is like expecting a four-cylinder car engine to turn over normally on three cylinders” (68). Further investigations are however needed to clarify the precise contribution of an altered striatal dendritic morphology to the genesis of LID (69).

## l-DOPA-Induced DA Release in the Dyskinetic Brain

PET imaging studies in PD patients have established a link between l-DOPA-induced motor complications and large fluctuations in striatal DA levels (20). In a seminal study using [^11^C] raclopride PET, De La Fuente Fernandez and coworkers compared the dynamics of striatal DA release between PD patients affected by LID and patients with a stable response to therapy (20). One hour after l-DOPA administration, dyskinetic patients exhibited significantly greater changes in striatal DA levels than did stable l-DOPA responders (21). Similar results were obtained by Piccini’s group, who also established a positive correlation between changes in striatal DA levels and severity of peak-dose LID (22). One limitation of these human studies is that the absolute extracellular concentrations of DA, hence their impact on changes in [^11^C] raclopride binding, were not accessible to investigation. This concern is relevant because the dyskinetic PD patients in these studies had a longer disease duration than did stable l-DOPA responders (21, 22). A longer disease duration may potentially lead to lower striatal DA levels at baseline.

Microdialysis studies in rodent models of LID have been very useful in clarifying the relationship between dyskinesia and absolute striatal DA concentrations “on” and “off” l-DOPA. In a seminal study, Meissner and colleagues compared striatal extracellular DA levels in 6-OHDA-lesioned rats exposed to a prior course of treatment with l-DOPA or saline (70). l-DOPA was given at a high dose (50 mg/kg/day per 10 days), which induced AIMs in all of the treated animals. A striking result of this study is that the same peripheral dose of l-DOPA elicited a larger increase in striatal extracellular DA levels in l-DOPA-primed animals compared to saline-treated ones (70). Other microdialysis studies were performed in 6-OHDA-lesioned rats that had been chronically treated with a lower dose of l-DOPA (6 mg/kg/day), upon which some of the animals remained free from AIMs. These studies reported larger striatal levels of l-DOPA (71) or DA (72, 73) in dyskinetic animals compared to non-dyskinetic cases. The most pronounced between-group difference in striatal DA levels occurred at the peak of the l-DOPA-induced surge, i.e., 40–60 min after l-DOPA administration. DA concentrations did not however differ between dyskinetic and non-dyskinetic animals either at baseline or at later time points post drug dosing (72, 73). Although dyskinetic animals showed a larger increase above baseline (ΔDA), their absolute DA concentrations never exceeded the values measured in intact control animals (72). Interestingly, a similar pattern of group differences was observed in the substantia nigra, which was monitored simultaneously with the striatum in one study (72).

Taken together, these results show that both ΔDA and absolute DA concentrations at the peak of the l-DOPA effect are larger in animals affected by involuntary movements compared to non-dyskinetic cases, despite similar baseline DA levels. The larger ΔDA values in dyskinetic rats are in keeping with the results of [^11^C] raclopride-PET studies in dyskinetic PD patients, though apparently at variance with other experimental data. In particular, a recent microdialysis study in the macaque model of LID has failed to detect a significant increase in striatal extracellular DA levels after l-DOPA administration, whereas striatal levels of DOPA showed a robust increase (74). According to the authors interpretation, these data indicate that a low DOPA decarboxylase activity in parkinsonian primates limits the production of DA from exogenous l-DOPA, differently from the situation encountered in 6-OHDA-lesioned rodents (74). These unexpected results prompt the interim reflection that the rat model of LID is more suitable than the macaque one to reproduce the presynaptic disturbances seen in the human condition. Indeed, [^11^C] raclopride binding is displaced by DA, and not by l-DOPA itself.

## Serotonin Neurons as an Aberrant Source of DA Release “on” l-DOPA

The first report implicating serotonin neuron as a source of DA release “on” l-DOPA was provided by Tanaka and colleagues (75). These authors compared extracellular DA levels in the striatum of 6-OHDA-lesioned rats that had sustained or not an additional chemical lesion of serotonin neurons. Rats in the double-lesion group exhibited a dramatic 80% reduction in l-DOPA-induced DA efflux (75). Another important early study used a similar approach to show that a serotonin lesion completely suppressed the induction of both rotational behavior and striatal c-Fos expression by l-DOPA in 6-OHDA-lesioned rats (76). The authors of these studies suggested that the action of l-DOPA in PD critically depends on its conversion to DA in serotonin neurons.

As explained above, serotonin neurons are endowed with the enzymes that convert l-DOPA to DA, and package this DA into synaptic vesicles. A double-labeling immunofluorescence study in rats treated with l-DOPA has indeed revealed immunoreactivity for DA in serotonin-positive dorsal raphe neurons and their striatal projections (77).

It is therefore hardly surprising that serotonin neurons become an important source of l-DOPA-derived DA release in a situation where nigrostriatal neurons are severely damaged. A relationship between LID severity, on one hand, and morphological or autoradiographic measures of striatal serotonin innervation, on the other hand, has been detected in both rat and non-human primate models of PD by several studies (78–81). These results fit well with our observation that chronically l-DOPA-treated rats with larger ΔDA values “on” l-DOPA show higher striatal levels of serotonin and its metabolite at baseline, suggestive of a denser 5-HT innervation (72).

Supporting the notion that 5-HT neurons release DA “on” l-DOPA, several studies in 6-OHDA-lesioned rats have shown that l-DOPA-induced peak DA efflux can be blunted by agonists of the serotonin autoreceptors, 5-HT1a and 5-HT1b (72, 82, 83). Agonists at these receptors dampen the activity of serotonin neurons, measured as either firing rate or neurotransmitter release (84). 5-HT1a and 5-HT1b receptor agonists have marked antidyskinetic effects in both rodent and non-human primate models of LID [reviewed in Ref. (3, 85)]. However, doses of 5-HT1a/b agonists that improve LID do not improve dyskinesias that are induced by apomorphine (86) or D1 receptor agonists (28). This pattern of effects indicates that low-medium doses of 5-HT1a and 5-HT1b agonists [cf. doses in (28, 72, 86)] interfere with presynaptic mechanisms of dyskinesia that are exclusively recruited by l-DOPA, not by dopaminergic agents acting directly on DA receptors. The efficacy of 5-HT1a and 5-HT1b agonists in reducing LID further indicates that DA release from serotonin neurons plays a causal role in LID. A compelling demonstration of this concept was provided by Carta and collaborators using selective lesions of 5-HT neurons (86). These lesions completely suppressed l-DOPA-induced AIMs in previously dyskinetic rats (86). Other studies applied a chemical lesion of 5-HT neurons to 6-OHDA-lesioned rats before treating them with l-DOPA, and demonstrated a positive association between the levels of residual striatal 5-HT innervation and the severity of dyskinetic movements induced by the treatment (87).

Why would DA release from 5-HT neurons be so prone to induce LID? Serotonin neurons lack presynaptic mechanisms that can sense and regulate their DA release, such as DA autoreceptors and DAT [reviewed in Ref. (15)]. Thus, in situations where both baseline DA levels and DAT activity are severely reduced (which is the case in advanced PD), DA release from serotonin neurons is bound to produce large swings in DA levels. Moreover, DA efflux from 5-HT neurons will be ectopic in terms of both subcellular release sites and anatomical distribution. Accordingly, an elegant microdialysis study in 6-OHDA-lesioned rats reported very large increases in DA levels “on” l-DOPA in many brain structures (including hippocampus and prefrontal cortex), and the increases were totally abolished by a complete lesion of serotonin neurons (88). These large extrastriatal DA surges induced by l-DOPA most likely contribute to the development of both motor and non-motor complications to therapy (89). With respect to LID, a recent study in the rat has linked the stimulation of cortical D1 receptors to the expression of involuntary movements through a local generation of high-frequency oscillatory activities (90).

## Debate on the Involvement of 5-HT Neurons in LID

Although the studies reviewed above are quite consistent, the concept that 5-HT neurons provide a major source of DA release in LID has met some resistance. Here follows a summary of common objections presented to us in the form of scientific correspondence. First, it is pointed out that the role of 5-HT neurons in LID has been studied in animals with relatively intact serotonin projections, which would be unlike the situation in the advanced stages of PD. Second, it is pointed out that a degree of striatal DA denervation as dramatic as in these animals would occur only in the very terminal stages of PD, implying that there would always be some nigrostriatal fibers ready to release DA in dyskinetic PD patients. Third, the comment has been put forward that astrocytes represent a much more abundant compartment than 5-HT projections to take up l-DOPA and convert it to DA in the striatum. All these objections are warranted, but also quite addressable with data available in the published literature.

As to the first point, post-mortem biochemical studies of 5-HT markers in PD have revealed that the loss of serotonergic innervation is more severe in the caudate than the putamen. In the latter structure, detectable levels of serotonergic markers persist until the terminal stages of PD (91). Accordingly, PET imaging studies in patients with advanced PD have detected only 30% reduction in putaminal serotonin transporter (SERT) binding (92), whereas, dopaminergic markers may be reduced by over 75% in the same structure (93). Post-mortem autoradiographic studies of SERT and DAT binding activities in the PD putamen are in keeping with the PET imaging investigations (42, 43, 80, 94). Furthermore, a post-mortem autoradiographic study has revealed larger SERT binding density in the post-commissural putamen in PD cases with LID compared to non-dyskinetic subjects (80).

Regarding the extent of DA denervation in the human disease, a recent pathological study has reported a virtual absence of DA fiber markers in the posterior putamen already at 4–5 years from PD diagnosis (95). Thus, the levels of DA denervation occurring in the dorsolateral striatum in animal models of LID are comparable to those in the post-commissural putamen (the motor part of the striatum) in mid-advanced stages of PD. And these are the stages where motor complications to therapy start to appear (cf. Figure 1).

As to the role of non-neuronal cells in handling l-DOPA, while this phenomenon certainly deserves further investigation (see below), it should be pointed out that neither glia nor vessel-associated cells have a capacity for vesicular storage and release of neurotransmitters. This is an important point, because microdialysis studies in 6-OHDA-lesioned rats have shown that l-DOPA-induced DA release is significantly reduced by reserpine, a VMAT blocker (96), and also by tetrodotoxin (TTX) (72, 97), a sodium channel blocker inhibiting the generation of action potentials. Thus, the bulk of DA efflux “on” l-DOPA has a neuronal origin even in animals with complete nigrostriatal DA lesions. Some authors have proposed that striatal interneurons expressing TH may provide a source of DA production and l-DOPA conversion in PD (98–100). However, it is as yet unclear whether these neurons can actually release DA [cf. (101)], and the expression of AADC in these cells appears to be very low, at least in rodents (41).

A proof-of-concept that 5-HT neurons release DA in patients affected by LID has been recently provided by Politis and coworkers using PET imaging techniques (102). In this study, dyskinetic PD patients were compared to patients with a stable response to therapy (“stable responders”) using both a SERT ligand ([^11^C]-DASB PET) and [^11^C] raclopride. In agreement with previous studies (see above), a standard dose of l-DOPA induced a larger displacement of [^11^C] raclopride binding in the dyskinetic group. Interestingly, the magnitude of [^11^C] raclopride displacement was positively correlated with the striatal levels of [^11^C] DASB binding, suggesting a relationship between peak DA efflux “on” l-DOPA and the density of striatal 5-HT innervation. Further to these observations, the authors evaluated the effects of buspirone, a compound with 5-HT1a agonistic activity, on the change in [^11^C] raclopride binding induced by l-DOPA administration. Intriguingly, buspirone reduced the magnitude of raclopride displacement only in dyskinetic PD patients, while having no effect at all in the stable responders. Furthermore, dyskinetic patients exhibiting a greater response to buspirone displayed a larger signal on the [^11^C] DASB PET scans, indicating larger striatal levels of serotonergic terminals. Finally, a strong positive correlation between AIM ratings and [^11^C] DASB binding density was found in the group of patients with peak-dose LID of mild-moderate severity (102). The authors concluded that striatal serotonergic terminals contribute to LID in human PD via aberrant processing of exogenous l-DOPA and release of DA as false neurotransmitter, quite in agreement with the results obtained in rat studies (102).

## Debate on the Plasticity of the Serotonin System in LID and Its Animal Models

The serotonin system is highly vulnerable to age-related degenerative changes, but also highly plastic (103–105). Functional and structural adaptations of the serotonin projections may therefore impact on their role in LID.

In many toxin-based animal models of PD, the neurotoxic lesion induces partial damage of ascending 5-HT projections, followed by a long-term compensatory sprouting of 5-HT axon fibers (81, 106–108). Furthermore, chronic dyskinesiogenic treatment with l-DOPA has a growth-promoting effect on serotonin axon terminals (78, 80, 81), which is likely dependent on the treatment-induced upregulation of BDNF (80). The treatment-induced sprouting of 5-HT axon terminals requires a previous severe DA denervation of the affected region, as well as a partial lesion of 5-HT afferents, as it does not seem to occur when LID is produced in animal models of PD having intact serotonin projections [cf. (109)].

The striking plasticity of the 5-HT system in animal models of PD–LID has raised concerns that the importance of this system may be overestimated in the experimental models relative to the human disease, because serotonin neurons are expected to degenerate, not to grow new axon terminals, in PD. However, in the study by Politis and coworkers (102), the dyskinetic patients with longest disease duration exhibited a remarkably preserved serotonin terminal function. Thus, striatal levels of [^11^C]-DASB binding did not differ between the severely dyskinetic patients and the subjects with a stable response to therapy, who had a significantly shorter disease duration (102). These results are at variance with the expected loss of [^11^C]-DASB binding during the progression of PD (92), and may in fact suggest that serotonin axon terminals mount a long-term sprouting response in human LID, analogous to that seen in the animal models. Further support to this interpretation comes from an autoradiographic study of SERT radioligand binding density in the human post-mortem putamen and pallidum, showing larger SERT binding levels in PD patients with clinical records of LID compared to non-dyskinetic cases (80). In this study, a linear correlation was found between SERT binding density and number of SERT-immunoreactive axonal varicosities, at least in the pallidum (80).

At variance with the evidence above, some recent studies in 6-OHDA-lesioned rats have suggested that chronic l-DOPA treatment may have deleterious effects on serotonin neurons. In one study, animals were treated with l-DOPA (12 mg/kg/day) for 28 days, after which tissue levels of DA and serotonin were measured in several brain regions at various intervals following the last l-DOPA dose (89). A reduced ratio between serotonin and DA concentrations occurred for up to 4 h post l-DOPA administration in all the structures examined. The authors concluded that l-DOPA treatment had increased DA levels while reducing 5-HT levels in all brain regions (89). These results may reflect the fact that DA displaces 5-HT from synaptic vesicles within serotonin axon terminals (77, 86). If serotonin is displaced from the vesicles, its degradation will be faster and its tissue contents reduced, at least for a few hours following the administration of l-DOPA. However, Eskow Jaunarajs and colleagues proposed that long-term l-DOPA therapy may be directly detrimental to serotonin neurons through mechanisms involving oxidative stress, an idea supported by some observations *in vitro* (89). Endorsing the above interpretation, a microdialysis study performed in rats previously treated with l-DOPA (12 mg/kg/day for 10 days) reported a lower magnitude of l-DOPA-induced DA efflux in several brain regions compared to that measured in acutely l-DOPA-treated animals (110). The authors concluded that chronic l-DOPA therapy negatively affects the functionality of serotonin neurons, at least if high drug doses are used (110). These results are, however, at variance with those reported by other studies using high doses of l-DOPA (70).

While the debate on the degeneration and plasticity of 5-HT neurons in PD–LID is still ongoing, there is agreement that 5-HT receptors in the brain show pronounced functional adaptations. In particular, increases in striatal and cortical levels of 5-HT1a and 5-HT1b receptors, as well as their adaptor proteins (111), have been reported by several studies performed in animal models of PD and LID [partially reviewed in Ref. (112)]. Further studies are needed to verify the occurrence of these adaptations in the human disease, and to clarify their functional consequences. For example, it is likely that these receptor adaptations may impact on the responsiveness to antidyskinetic treatments targeting 5-HT1a and 5-HT1b receptors.

## Gliovascular Mechanisms

In addition to high DA levels, dyskinetic animals show a large increase in the extracellular levels of l-DOPA following peripheral drug administration (71, 74, 113). A study in non-human primates has even suggested that l-DOPA does not need to be converted to DA in order to elicit AIMs (74).

The concentrations of l-DOPA in the brain extracellular fluid reflect the balance between drug entry and drug uptake/metabolism by brain cells. There are no indications that the uptake of l-DOPA by brain cells is impaired in dyskinetic animals, and it is therefore warranted to ask whether its entry could be increased. l-DOPA enters the brain from the blood stream via the L-type amino acid transporter system present in endothelial cells of the blood–brain barrier (BBB) (114, 115). Thus, the passage of l-DOPA from blood to brain will depend on the same variables that regulate the extraction of any substance, that is: (1) capillary permeability, (2) the capillary surface area, and (3) the regional blood flow (116). In the case of l-DOPA, a fourth variable should be considered, namely, the possibility of an active drug metabolism at the capillary level.

**Figure 5:**
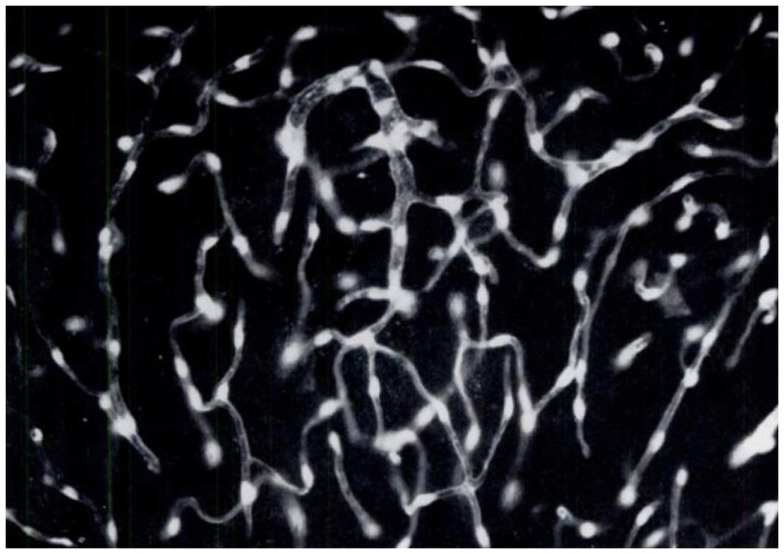
**Brain endothelial cells and pericytes produce dopamine following systemic administration of l-DOPA**. In the 60s, a group of Swedish pharmacologists led by E. Rosengren discovered that brain endothelial cells and pericytes are a significant site of dopamine production following treatment with l-DOPA. This photomicrograph represents a section of rat cerebellum processed for the Falck–Hillarp catecholamine histofluorescence method to visualize DA-containing cells. The rat had received an injection of l-DOPA (50 mg/kg, combined with the monoamine-B inhibitor nialamide) shortly before being killed. The authors commented, “It was evident that the fluorescent material occurred throughout the capillary walls giving almost a three-dimensional appearance of the capillary tubes. Fluorescence of high intensity (was found) in cytoplasm and nucleus of both endothelial cells and pericytes” [Reproduced with permission from Ref. (51)].

Already in the 60s, studies based on the Falck–Hillarp catecholamine histofluorescence method had indicated that brain capillaries critically regulate the entry of l-DOPA into the brain parenchyma (51). Endothelial cells and pericytes were revealed to be the first site of l-DOPA uptake, conversion, and metabolism in the brain (Figure 5), and were found to express very high levels of both AADC and monoamine oxidase B (51). It was thus proposed that cells lining cerebral microvessels form an enzymatic barrier to the entry of l-DOPA (51). Further to these studies, it was recently reported that l-DOPA accumulates not only in the microvessels, but also in astrocyte cell bodies and astrocytic endfeet surrounding cerebral microvessels (117).

Endothelial cells, pericytes, and perivascular astrocytes form a functional unit that controls both capillary permeability and regional cerebral blood flow (rCBF) [reviewed in Ref. (118, 119)]. Both of these parameters are dynamically regulated in the brain to match the metabolic activity of neurons, and this process (termed “neurovascular coupling”) is modulated by monoaminergic afferents that innervate cerebral arterioles and microvessels (120–124).

Interestingly, while regional glucose metabolism (which is mainly driven by neuronal activity) and rCBF are well-matched in PD patients during the “off” medication state, the administration of l-DOPA greatly increases rCBF without elevating glucose metabolism in a brain network that includes putamen, pallidum, and midbrain–pons (125). In this brain network, the dissociation between flow and metabolism is particularly striking in patients affected by LID (125). These findings suggest that l-DOPA exerts hemodynamic effects that are independent of its modulation of neuronal metabolic activity, thus superseding physiological mechanisms of neurovascular coupling in the affected brain regions. A similar phenomenon occurs in the rat model of LID, which features a large increase in rCBF “on” l-DOPA in many parts of the basal ganglia, often in the absence of large concomitant changes in glucose metabolism (126).

The flow-metabolism dissociation response is a particularly intriguing phenomenon as it may signal a previously overlooked effect of l-DOPA on gliovascular cells (126). Moreover, this phenomenon may result in higher extracellular levels of l-DOPA in dyskinetic subjects (125, 126), impacting on the presynaptic mechanisms of LID. The underlying mechanisms are however unclear. Evidence of flow-metabolism dissociation has thus far been found only in specific regions, and the observed regional pattern cannot be readily explained by regional differences in either DA efflux “on” l-DOPA (88) or gliovascular expression of DA receptors (43, 120, 122, 127). Some interesting mechanistic suggestions have however emerged from studies performed in the rat model of LID. In 6-OHDA-lesioned rats treated with l-DOPA, regions with large increases in blood flow “on medication” exhibit endothelial proliferation and angiogenic activity when the treatment is given chronically (126). Furthermore, some of these regions exhibited an increased microvascular density and upregulation of angiogenesis markers in a post-mortem study of basal ganglia tissue from dyskinetic PD patients (43). These findings suggest that the large increases in rCBF “on” l-DOPA and the angiogenic response to the chronic treatment are interrelated phenomena, which are critically regulated by gliovascular cells in the affected brain regions (126). Investigating this hypothesis is likely to yield important insights into previously overlooked neurovascular effects of l-DOPA, uncovering novel therapeutic targets.

## Changes in BBB Permeability: The Findings and the Debate

As mentioned above, capillary permeability is one of the factors determining the central availability of l-DOPA. The BBB is a selective diffusion barrier that relies on specialized properties of the brain’s capillary endothelium, such as the presence of tight cell–cell junctions, low levels of pinocytotic activity, and the expression of selective transporter proteins at the plasma membrane [reviewed in Ref. (128)]. Several independent studies suggest that the functionality of the BBB becomes impaired during the progression of PD (129–131). For example, the ratio between albumin concentrations in cerebrospinal fluid (CSF) and plasma is increased in PD patients with advanced disease compared to age-matched controls (131). Interestingly, higher albumin ratio values were measured in patients receiving DA replacement therapy compared with untreated subjects (131).

It has been suggested that the neuroinflammation associated with neurodegeneration leads to an increased BBB permeability due to the vascular effects of proinflammatory cytokines [see Discussion in Ref. (132), and references therein]. However, while neuroinflammation is a widespread finding in PD (133), the permeability problem appears to depend on focal areas of BBB dysfunction within the striatum and the midbrain. These areas show signs of angiogenic activity (43, 126, 132, 134). Several studies in both parkinsonian animals and human PD have indeed detected endothelial proliferation and other markers of active angiogenesis within the substantia nigra and the striatum (43, 134–137). Because active angiogenesis entails a transient increase in vessel permeability, it will inevitably lead to a localized leakage of the BBB when it occurs in the brain (138). Accordingly, studies in rat models of PD have revealed localized leakage of BBB tracer molecules (132) or downregulation of BBB proteins (139) precisely on vessels having angiogenic features.

When treatment with l-DOPA produces dyskinesias, it may aggravate the BBB dysfunction associated with PD, or even induce a new pattern of dysfunction. In the rat model of LID, dyskinetic animals exhibit endothelial proliferation, increased BBB permeability, and upregulation of vascular endothelial growth factor (VEGF) in the lateral striatum and the basal ganglia output nuclei (the substantia nigra pars reticulata and the entopeduncular nucleus, i.e., rodent equivalent of the GPi) (43, 139–141). These phenomena only occur on the DA-denervated side of the brain, and they are positively associated with the development of LID (139, 141). l-DOPA induces this angiogenic activity via stimulation of D1 receptors and activation of ERK1/2 signaling (140). Treatments that antagonize VEGF attenuate the gradual increase in dyskinesia severity during a chronic course of l-DOPA administration (43, 141), while inhibiting the angiogenic activity and BBB dysfunction induced by l-DOPA in the basal ganglia (43). Along with human pathological observations (43, 137), these findings suggest that a treatment-induced, VEGF-dependent angiogenic activity in the basal ganglia contributes to an aggravation and chronicization of LID in the advanced stages of PD (43).

The pathophysiological implications of these findings are, however, poorly understood. We have proposed that the increased BBB permeability associated with angiogenesis may contribute to an increased entry of l-DOPA in the affected regions (i.e., the motor part of the striatum and the basal ganglia output nuclei) (139). Supporting this proposition, dyskinetic animals were found to exhibit increased striatal and nigral uptake of an intravenous tracer molecule (which normally does not cross the BBB) having a molecular weight similar to l-DOPA (126). Importantly, leakage of this tracer into the striatal parenchyma was detected at significant levels at 60 min, but not 24 h after the administration of l-DOPA (126). This observation is interesting because it suggests an association between increased rCBF “on” l-DOPA and BBB hyperpermeability in dyskinetic subjects (126). In other words, the high rCBF associated with LID (125, 126) would cause BBB leakage at the level of immature microvessels, which form in the striatum and its output nuclei because of the combined effect of DA denervation and chronic l-DOPA treatment (126). In keeping with this suggestion, an increased perfusion has been shown to enhance tight-junction opening between endothelial cells in other models of brain disease involving angiogenesis or microvascular pathology (142). Further investigations are needed to clarify the relative importance of an increased BBB permeability in producing high extracellular levels of l-DOPA in LID.

The suggestion that BBB permeability is enhanced in LID has raised some debate (143). It is often argued that the peripheral DOPA decarboxylase inhibitors included in standard l-DOPA preparations [i.e., carbidopa or benserazide, reviewed in Ref. (3, 4)] are unlikely to enter the brain. If they did, the treatment would not engender an increase in central levels of DA, whereas raclopride–PET studies unequivocally demonstrate striatal DA release after the administration of l-DOPA to PD patients. However, studies in both intact and 6-OHDA-lesioned rats indicate that peripheral DOPA decarboxylase inhibitors significantly reduce central AADC activity only at doses much higher than those given to patients (144, 145). More importantly, doses of benserazide reducing striatal AADC activity by over 50% did not have any effect on either basal DA levels or l-DOPA-induced DA release in the striatum (145, 146). To achieve a significant effect on the above parameters, benserazide had to be administered at the dose of 50 mg/kg, which reduced striatal AADC activity by ≥80% (145, 146). Such a dose is manifold larger than the highest benserazide dosage to which a PD patient will ever be exposed. In a study using 6-OHDA-lesioned rats, not even 50 mg/kg benserazide had any significant effect on the increase in extracellular DA levels induced by l-DOPA, affecting only the time to reach the peak (145).

## Role of Noradrenaline Neurons

Dopamine is the immediate precursor of NA along the catecholamine biosynthetic pathway, and extracellular NA levels increase in the DA-denervated striatum after a peripheral injection of l-DOPA. Interestingly, this increase is significantly larger when the treatment induces involuntary movements (73). An elevation in striatal NA levels has been suggested to contribute to LID because local infusions of NA in the DA-denervated striatum induce AIMs in the rat (73, 147). Based on these findings, one would expect LID to be improved by lesions of central NA projections. Quite in contrast with this prediction, most studies addressing the impact of noradrenergic denervation on LID have reported a worsening of dyskinesia, which was due either to an increased peak severity (148, 149) or to an increased duration of the involuntary movements (150). Other studies have not, however, detected a significant worsening of LID, even when the noradrenergic denervation resulted in a worsening of motor and cognitive deficits (151, 152). These apparent discrepancies are likely to depend on technical differences regarding NA lesion procedures and/or types of 6-OHDA models used in different studies. In this regard, it is useful to know that injections of 6-OHDA in the medial forebrain bundle (MFB) damage also ascending NA fibers, an effect that cannot be completely prevented by pretreating animals with blockers of NA uptake, such as desipramine (unpublished data by the Cenci’s lab). Thus, a large 6-OHDA lesion in the MFB may occlude the effect of a subsequent NA lesion, even more so if the latter is applied using toxins that damage NA projections but leave their cell bodies intact (150).

Despite the above discrepancies, a large amount of data point to an involvement of the NA system in the motor complications of PD therapy. This system is highly vulnerable to the neurodegenerative process in PD (153) and to the neurotoxins that are used to create PD models in animals [reviewed in Ref. (30)]. Moreover, treatment with l-DOPA appears to modulate the activity of brain NA neurons, as indicated by changes in NA cell firing in the locus coeruleus region, and by an increased NA efflux in their projection targets (73, 150). That the NA system in causally involved in LID is suggested not only by the results of lesion studies in the rat (148–150), but also by a vast pharmacological literature investigating the effects of NA receptor modulators.

Several studies in rat and primate models of PD have indeed shown that modulators of NA receptors improve LID. Many studies have evaluated antagonists of α_2B/C_-adrenoceptors, and found that they reduce the severity of l-DOPA-induced AIMs, and that they also can prolong the anti-akinetic effect of single l-DOPA doses (154–158). One potential underlying mechanism may involve a reduction of peak extracellular levels of both DOPA and DA, which the α_2C_ adrenoceptor antagonist idazoxan has been shown to achieve at a dose that significantly reduces the severity of LID (113). The mechanisms by which central NA neurons modulate the effects of l-DOPA remain, however, poorly understood. Given that the NA system has widespread modulatory functions in the brain, these mechanisms are bound to be very complex. Relevant to the presynaptic mechanisms of LID are the modulatory effects of NA on several afferent striatal systems, including 5-HT and DA axon terminals (159–161), and the key role of locus coeruleus neurons in regulating both cerebral blood flow and capillary permeability (124, 162), and in maintaining the integrity of the BBB (163).

## Concluding Remarks

l-DOPA remains the most effective treatment for PD and understanding how this drug is handled by, and in turn affects, a parkinsonian brain, is an undisputed research priority, not least for the sake of developing better treatment options.

In the past few years, research on the presynaptic mechanisms of LID has generated results of great translational importance, but also scientific controversy. In this article, I have reviewed both the findings and the controversies, while highlighting important aspects that call for further investigations.

Some of the concepts presented in this article are, however, quite uncontroversial and have already inspired a clinical development of new treatments. Thus, the concept that large swings in striatal DA levels are the culprit behind motor fluctuations and dyskinesia has prompted the development of new methods of continuous l-DOPA delivery, which are now available in several countries [reviewed in Ref. (3)]. While these therapies have a proven efficacy against the motor fluctuations (164), the extent to which they can eliminate already established dyskinesias remains to be demonstrated.

The concept that LID depends on DA release from serotonin neurons has raised both interest and discussion. That 5-HT neurons can produce and release DA “on” l-DOPA is now widely accepted. A debate, however, persists regarding the relative importance of this phenomenon. PD dyskinesias are conceivably more complex than the models of peak-dose LID obtained in animals with “clean” nigrostriatal lesions. For example, in the advanced stages of PD, the involuntary movements may exhibit a variable and unpredictable relationship with the timing of drug administration, and they may be induced by dopaminergic agents that do not release any DA in the brain. A point of recent discussion pertains to the role of DA release from 5-HT neurons in inducing involuntary movements as opposed to “good” anti-akinetic effects. Two recent studies (165, 166) have suggested that DA release from serotonin neurons not only generates dyskinesia but may also mediate the therapeutic benefit of l-DOPA. An implication of these findings is that antidyskinetic treatments based on the stimulation of 5-HT_1A/B_ receptors (dampening transmitter release from 5-HT neurons) may have an unfavorable risk-benefit profile in the advanced stages of PD, when most l-DOPA-derived DA release is likely to come from 5-HT neurons, at least in the motor regions of the striatum. Accordingly, large clinical trials of 5-HT_1A_ receptor agonists in LID appear to have faced some difficulties in defining a suitable therapeutic window for the investigational drugs [reviewed in Ref. (3)]. It should be noted, however, that the 5-HT_1A_ ligands so far evaluated in PD patients had partial agonist activity and many off-target effects. To really appreciate the potential of this strategy, it will therefore be important to test more potent and selective compounds.

During the past few years, we have learned that l-DOPA pharmacotherapy affects not only neurons, but also microvascular (43, 125, 126, 141) and glial compartments (43, 117, 167) within the basal ganglia and the midbrain. Findings obtained in rat models of LID have revealed a previously unappreciated plastic potential of basal ganglia microvessels, sparking a new interest in the effects of dopaminergic medications on the neurovascular unit. This topic clearly deserves further investigation. An emerging research is uncovering orchestrated actions of gliovascular cells, immune cells, and neurons in the maladaptive plasticity associated with brain diseases and their treatments (168–171). Investigating the interactions between neuronal and gliovascular compartments is therefore required to fully understand the long-lasting plasticity at the basis of LID. Such an understanding will make it possible to devise new preventive strategies. Ultimately, preventive interventions may represent the best approach to this medical problem because, once established, LID is probably impossible to completely eliminate with add-on pharmacological treatments.

## Conflict of Interest Statement

The author declares that the research was conducted in the absence of any commercial or financial relationships that could be construed as a potential conflict of interest.
